# Mineralocorticoid Receptor Antagonists in Heart Failure with Preserved Ejection Fraction: A Systematic Review and Meta-Analysis

**DOI:** 10.3390/jcm14103598

**Published:** 2025-05-21

**Authors:** Mithila Zaheen, Fardin Ferdous, Anjalee T. Amarasekera, Johannes Petutschnigg, Frank Edelmann, Timothy C. Tan

**Affiliations:** 1 Department of Cardiology, Blacktown Hospital, Western Sydney Local Health District, Sydney, NSW 2148, Australia; 2 University of Sydney, Sydney, NSW 2006, Australia; 3 Western Sydney University, Sydney, NSW 2751, Australia; 4 Department of Cardiology, Westmead Hospital, Western Sydney Local Health District, Sydney, NSW 2145, Australia; 5 Westmead Applied Research Centre (WARC), Faculty of Medicine and Health, University of Sydney, Sydney, NSW 2145, Australia; 6 Deutsches Herzzentrum der Charité, Klinik für Kardiologie, Angiologie & Intensivmedizin, Augustenburger Platz 1, 13353 Berlin, Germany; 7 German Centre for Cardiovascular Research (DZHK), Partner Site Berlin, Berlin Institute of Health (BIH), 13353 Berlin, Germany; 8 University of New South Wales, Sydney, NSW 2052, Australia

**Keywords:** HFpEF, diastolic heart failure, heart failure pharmacotherapy, echocardiography, exercise capacity, quality of life

## Abstract

**Background/Objectives**: Heart failure with preserved ejection fraction (HFpEF) is a complex clinical syndrome with limited therapeutic options. Mineralocorticoid receptor antagonists (MRAs) have been shown to improve clinical outcomes in patients with heart failure with reduced ejection fraction (HFrEF), but their use in patients with HFpEF remains controversial. The aim of this review is to evaluate whether the use of MRAs improves diastolic function, functional capacity, and quality of life in patients with HFpEF. **Methods**: A systematic literature search of scientific databases was performed to identify studies comparing the use of MRAs to placebo or no treatment in adult patients with HFpEF (2000–2024; English; PROSPERO registration CRD42022300783). Data were meta-analysed using a random-effects model for overall effect size measured as the standardised mean difference. **Results**: Pooled data revealed a significant benefit of MRA use compared to the control in decreasing E/e’ (SMD −0.21; 95% CI: −0.33 to −0.10, *p* = 0.00), with greater improvement seen with longer duration of treatment. A substantial reduction in systolic blood pressure (SMD −0.27; 95% CI: −0.53 to −0.02, *p* = 0.03) and diastolic blood pressure (SMD −0.18; 95% CI: −0.32 to −0.04, *p* = 0.01) was also noted. There was no significant difference in the 6 min walk distance, peak exercise capacity, or quality-of-life measures. Adverse events such as hyperkalaemia and worsening renal function were frequently reported in the MRA group. **Conclusions**: MRAs improve echocardiographic parameters of diastolic function and BP control; however, this did not translate into clinical outcomes of improved functional capacity or quality of life.

## 1. Introduction

**Figure 1:**
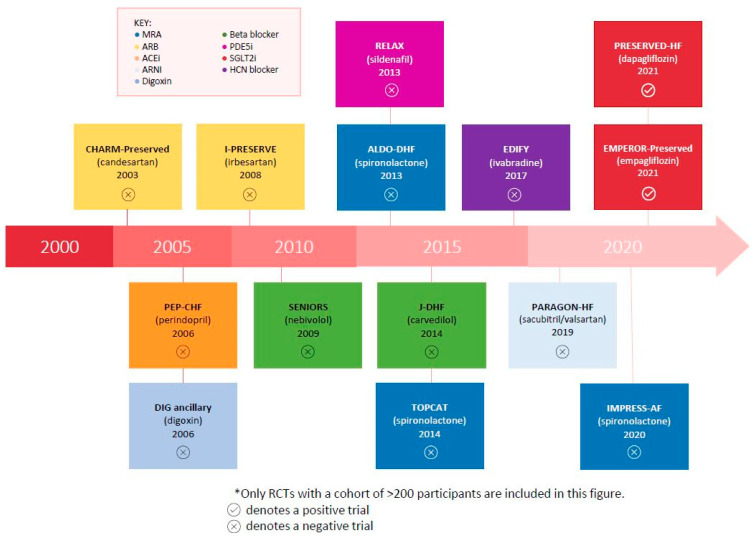
* Timeline of clinical trials for the pharmacological management of HFpEF.

Heart failure with preserved ejection fraction (HFpEF), previously known as diastolic heart failure, is an increasingly common condition that affects millions of people worldwide [1]. It is estimated that HFpEF accounts for more than half of all heart failure (HF) hospital admissions [1,2]. Despite its prevalence, there are limited evidence-based therapeutic options [1]. The majority of large randomized controlled trials (RCTs) for common HF therapies have been unsuccessful in meeting their primary end points in patients with HFpEF (Figure 1). Currently, there is evidence from recent clinical trials that sodium–glucose cotransporter 2 inhibitors have proven beneficial in HFpEF management [3,4,5]. However, aside from this, guideline-directed drug therapy for HFpEF is limited, and the mainstay of treatment involves hypertension management, fluid balance control, and optimisation of comorbidities [1].

Aldosterone is a mineralocorticoid hormone produced by the adrenal cortex [6]. In addition to its well-known effects in maintaining sodium and fluid balance, aldosterone has been shown to have an important role in the pathogenesis of HF [6,7]. Studies have demonstrated that aldosterone stimulates myocardial fibrosis and vascular stiffening, resulting in cardiac remodelling and diastolic dysfunction [6,7,8]. Aldosterone blockade has thus been proposed as a potential therapeutic target for patients with HF [6,7].

Mineralocorticoid receptor antagonists (MRAs), such as eplerenone and spironolactone, are diuretics that oppose the action of aldosterone at mineralocorticoid receptors [7]. Given that the use of eplerenone in rat models has been associated with attenuation of left ventricular diastolic dysfunction and reduction in myocardial fibrosis, the use of MRAs for the treatment of HFpEF in human patients appears promising [6,7,8].

While there is emerging evidence to support the role of MRAs in the treatment of heart failure with reduced ejection fraction (HFrEF), the benefits of the use of MRAs in patients with HFpEF are unclear [1,2,5]. The purpose of this review is to critically appraise and evaluate RCTs that address outcomes of diastolic function, functional parameters, quality of life (QoL), and safety of MRA use in patients with HFpEF.

Recent interest has also focused on finerenone, a non-steroidal selective MRA, which has demonstrated cardiovascular and renal benefits in patients with type 2 diabetes and chronic kidney disease (CKD)—a population with a significant overlap with HFpEF. The FIDELIO-DKD and FIGARO-DKD trials showed that finerenone reduced the risk of cardiovascular events and slowed CKD progression in high-risk patients, some of whom met contemporary HFpEF criteria [9,10]. However, these trials did not specifically assess the echocardiographic markers of diastolic function, which precluded their inclusion in this meta-analysis. Nonetheless, the emerging role of finerenone underscores the evolving therapeutic potential of MRAs in patients with preserved ejection fraction phenotypes [9,10].

## 2. Methods

### 2.1. Literature Search

A comprehensive literature search of online databases, including MEDLINE, EMBASE, CINAHL, CENTRAL, Science Direct, Scopus, and ProQuest, was undertaken in accordance with the Preferred Reporting Items for Systematic Reviews and Meta-Analysis (PRISMA) guidelines [11]. The key search terms are listed in Supplement S1. The literature search was restricted to the period from 1 January 2000 onwards due to concerns regarding the risk of inconsistency in the clinical diagnosis of HFpEF prior to 2000. The reference lists from the included studies and previous systematic reviews were hand-searched for additional studies. Google Scholar was manually searched for available grey literature and other relevant publications. The search was limited to studies in human subjects and the English language.

Two reviewers (M.Z. and F.F.) independently performed the initial screening of the titles. Full-text publications were reviewed separately and assessed for eligibility for inclusion. Any disagreements were resolved through mutual discussions and consensus with a third reviewer (A.A.).

### 2.2. Study Eligibility and Inclusion Criteria

Eligible studies were RCTs that compared MRAs with placebo or no treatment in adults (aged ≥18) with a diagnosis of HFpEF. HFpEF was defined as having clinical signs or symptoms of HF and a left ventricular ejection fraction (LVEF) ≥ 45%, or evidence of diastolic dysfunction on echocardiogram. Diastolic dysfunction was defined based on the criteria applied within each included study, which varied according to the guidelines in place at the time of the study. Commonly used parameters included the E/A ratio, E/e’, deceleration time (DT), and tissue Doppler imaging indices. Inclusion was not restricted to a specific grade or severity of diastolic dysfunction; studies that enrolled patients across any grade of diastolic dysfunction, or that did not stratify by grade, were included, provided the broader HFpEF definition was met. The included studies were required to report at least one outcome of interest. The primary outcome was echocardiographic parameters of diastolic function (E/A ratio, E/e’, EDT, LVMi, and LAVi). Secondary outcomes included systolic blood pressure (SBP), diastolic blood pressure (DBP), functional parameters (6 min walk distance [6MWD], peak exercise capacity [peak VO_2_], or New York Heart Association [NYHA] class), and QoL parameters (Minnesota Living with Heart Failure Questionnaire [MLWHFQ] or Kansas City Cardiomyopathy Questionnaire [KCCQ]). Exclusion criteria included studies that compared MRA to another intervention, studies with healthy persons enrolled in the control group, studies that did not report outcomes of interest, non-English publications, editorials, conference article proceedings, review articles, and studies describing animal models.

### 2.3. Data Extraction

Data extraction was undertaken by two reviewers (M.Z. and F.F.) and subsequently cross-validated. A standardised data collection template was used to collect the following from each study: year of publication, sample size, cut-off LVEF for HFpEF, characteristics of study participants (such as age, sex, comorbidities), intervention (including MRA and dose), control group (placebo or usual therapy), duration of follow-up, adverse events, outcome data, and associated commentaries. The presence of clinical heterogeneity was analysed from the data collection tables and compared by two reviewers.

### 2.4. Risk of Bias

The risk of bias within selected studies was independently assessed by two reviewers (M.Z. and F.F.), who were trained in using the Joanna Briggs Institute of Critical Appraisal Tools Checklist for RCTs [12]. This included an assessment of appropriate randomisation, allocation concealment, blinding, attrition rates, and selective outcome reporting. The overall risk of bias was categorised as low, high, or unclear.

### 2.5. Statistical Analysis

Data were represented in the form of a systematic review, forest plots, and meta-analyses using STATA Version 16.1. The mean, standard deviation, and standardised mean difference (SMD) with a 95% confidence interval (CI) were used for continuous data. When not available, the reviewers derived these results from confidence intervals in accordance with the Cochrane Handbook for Systematic Reviews of Interventions [13].

Missing data were observed across included studies in the form of attrition rates, losses to follow-up, and withdrawals. The original investigators were contacted by electronic correspondence to request further information on missing data. Missing data could either not be provided by the investigators or could not be obtained due to unsuccessful contact attempts. The reviewers critically appraised the potential impact of missing data on the overall results of the systematic review in the Discussion section.

Statistical heterogeneity was assessed visually by examining the distribution of results across forest plots for different outcomes. Statistical heterogeneity was quantified using the I^2^ statistic, which describes the percentage of variability in the effect estimates that can be attributed to heterogeneity as opposed to sampling error [13].

Due to the small sample sizes of the included studies and variability in study design, meta-analyses were performed using random-effects models in order to account for the anticipated clinical and methodological heterogeneity between studies. Although hypotheses to explore heterogeneity had been specified a priori (studies with high versus low percentage of women, studies with and without concurrent use of renin–angiotensin–aldosterone system [RAAS] inhibitors and use of selective versus non-selective MRAs), subgroup analyses were not considered feasible due to the small number of studies. The reviewers planned to undertake sensitivity analyses to examine the effects of including studies with an unclear risk of bias; however, there were insufficient data.

Reviewers undertook a comprehensive search of unpublished studies, abstracts, and grey literature in various conference databases to determine the risk of publication bias and selective reporting across the included studies. Publication bias was also assessed by examining the asymmetry of funnel plots.

## 3. Results

### 3.1. Study Selection

**Figure 2:**
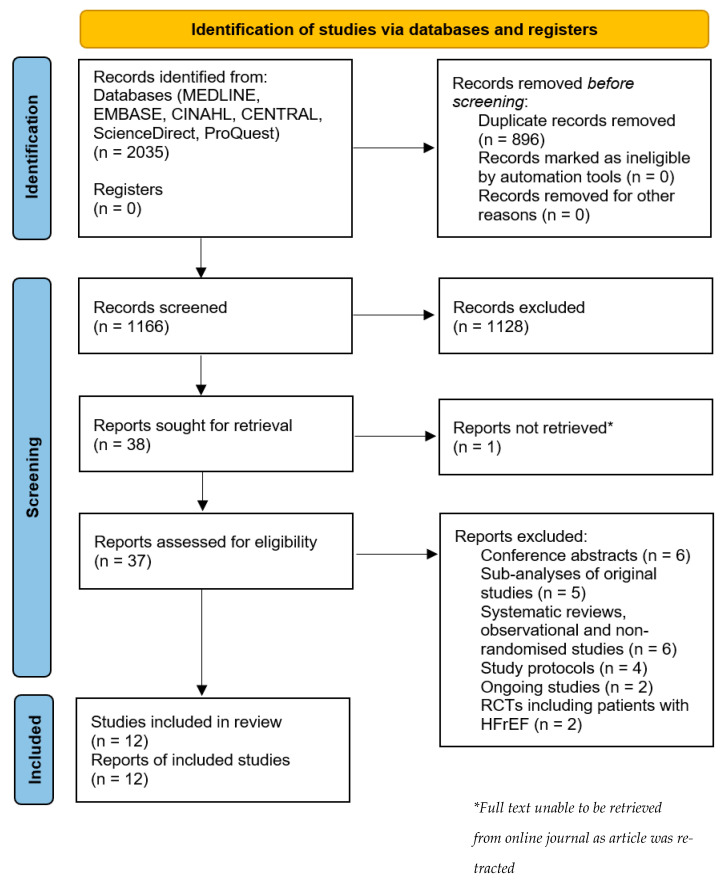
PRISMA 2020 flow diagram for new systematic reviews, which included searches of databases and registers only.

The initial search identified 2035 studies from 7 databases. After removal of duplicates, screening of titles and abstracts, and adding studies from previous reviews, 38 articles were considered highly relevant and selected for full-text review. Of these, 25 studies were excluded for various reasons: 6 articles were conference abstracts, 5 articles were sub-analyses of original studies, 6 studies were not RCTs (either systematic reviews or observational and non-randomised studies), 4 articles were study protocols, 2 studies were currently ongoing, and 2 articles were RCTs that did not include the population of interest. Ultimately, 12 studies met the inclusion criteria, and 11 of these were amenable for inclusion in the meta-analysis, randomizing a total of 1390 adults [14,15,16,17,18,19,20,21,22,23,24,25,26,27]. It should be noted that the study carried out by Shah et al. reported on diastolic function in a subgroup in the original study by Pitt et al. [20,27]. A PRISMA flow diagram that details the study selection process is presented in Figure 2.

### 3.2. Study Characteristics

A total of 12 RCTs were included in the qualitative analysis and 11 RCTs in the quantitative analysis. Two studies used eplerenone and ten studies used spironolactone. The dose of spironolactone ranged between 15 mg daily and 45 mg daily between studies, with the most common dose being 25 mg daily. Ten trials were placebo-controlled, and the control groups in two studies were untreated. The mean age of the participants was 70 years. The follow-up time of treatment ranged from four months to two years. Overall, the female-to-male ratio across the studies differed significantly. One study had an exclusively female study population, and four studies had a significant female to male ratio of greater than 80%. Five studies had an even female-to-male ratio, and four studies had a predominantly male study population of greater than 75%. The total sample size between the studies ranged from 30 to 250 participants.

The cut-off LVEF for HFpEF differed between studies: three studies included participants with an LVEF ≥ 45, eight with ≥50, and one with ≥55. All studies reported at least one measure of diastolic function on echocardiography, and one study measured diastolic function using cardiac MRI [21]. There was variable reporting of functional parameters such as 6MWD, peak VO_2_, and NYHA class. QoL measures were infrequently reported. Table 1 summarises the baseline characteristics of the included studies. An extended summary table can be found in Supplementary Materials.

### 3.3. Quality Assessment and Risk of Bias

Out of the 12 included studies, four were determined to have an unclear risk of bias due to a lack of clarity regarding allocation concealment and appropriate blinding of participants and investigators. Only four studies reported a true computer-generated randomisation process. The reasons behind losses to follow-up and associated implications were inadequately described in five studies. No protocol deviations were observed, and appropriate statistical analysis was used throughout all included studies. After reviewing funnel plots, unpublished papers, and grey literature, it was determined that there was a low risk of publication bias across studies. The assessment of risk of bias can be found in Table S2 in Supplementary Materials.

### 3.4. Diastolic Function

At least one echocardiographic parameter of diastolic function was reported in each included study. The most commonly reported parameters were the early mitral inflow velocity and the mitral annular early diastolic velocity ratio (E/e’) and E/A ratio, which were reported in nine included trials. Deceleration time (DT) was reported in eight studies.

**Figure 3:**
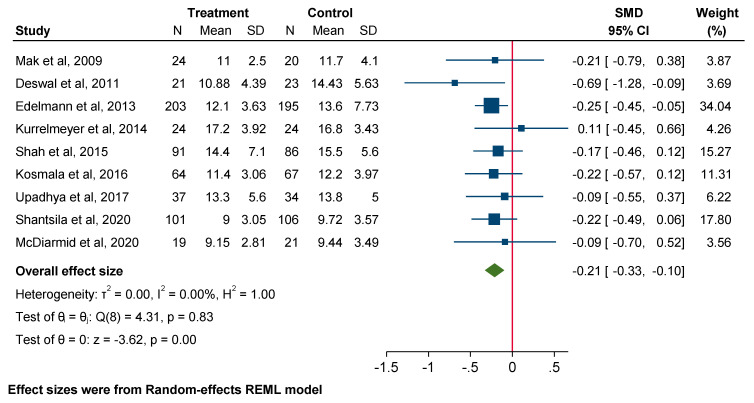
Forest plot for echocardiographic parameters: E/e’.

**Figure 4:**
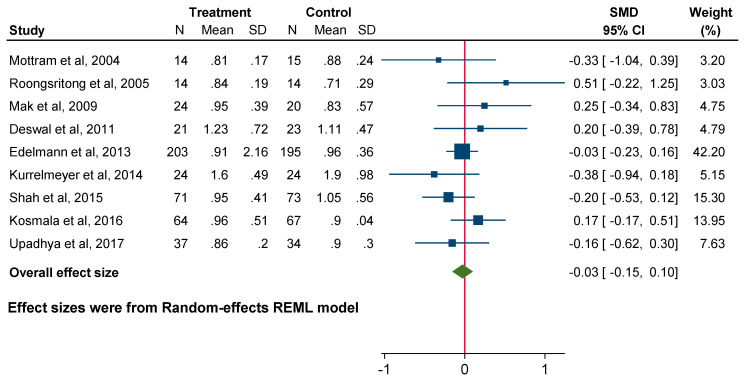
Forest plot for echocardiographic parameters: E/A ratio.

**Figure 5:**
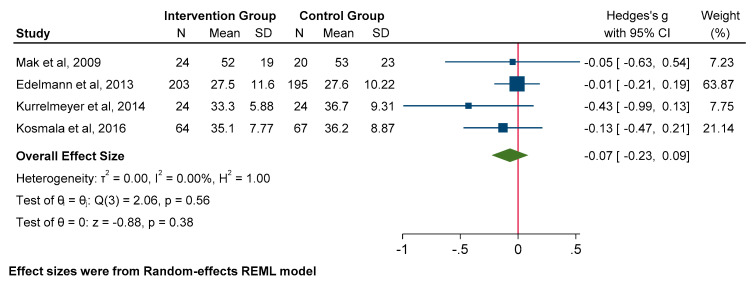
Forest plot for echocardiographic parameters: LAVi.

Pooled data revealed a significant influence of MRA use on decreasing E/e’ (SMD −0.21; 95% CI: −0.33 to −0.10, *p* = 0.00). In contrast, MRA use did not significantly affect the E/A ratio (SMD −0.03; 95% CI: −0.15 to 0.10, *p* = 0.68) or left atrial volume index (LAVi) (SMD −0.07; 95% CI: −0.23 to 0.09, *p* = 0.38) (Figure 3, Figure 4 and Figure 5).

**Figure 6:**
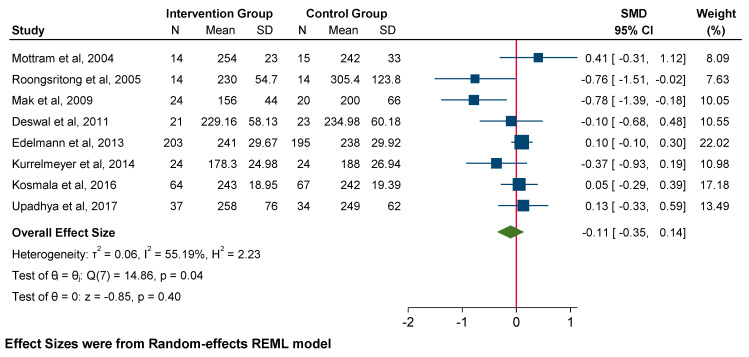
Forest plot for echocardiographic parameters: DT.

There was no significant difference in DT (SMD −0.11; 95% CI: −0.35 to 0.14, *p* = 0.40) or left ventricular mass index (LVMi) (SMD −0.01; 95% CI: −0.37 to 0.35, *p* = 0.95). However, it should be noted that there is potentially a moderate to high degree of heterogeneity within these measures (*p* = 0.04, I^2^ = 55% and *p* = 0.02, I^2^ = 76%, respectively). A subgroup analysis was unable to be undertaken due to insufficient data (Figure 6).

### 3.5. Effect on Blood Pressure

**Figure 7:**
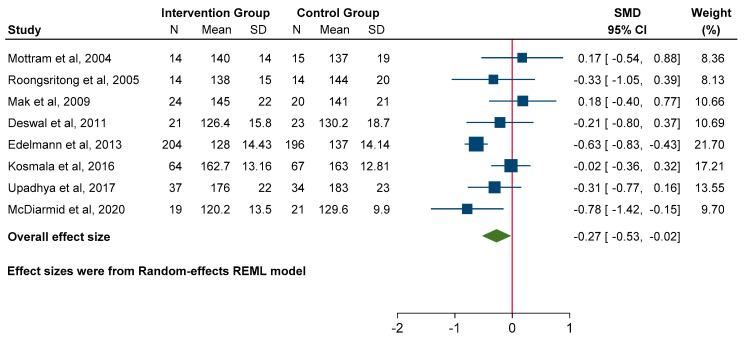
Forest plot for systolic blood pressure.

**Figure 8:**
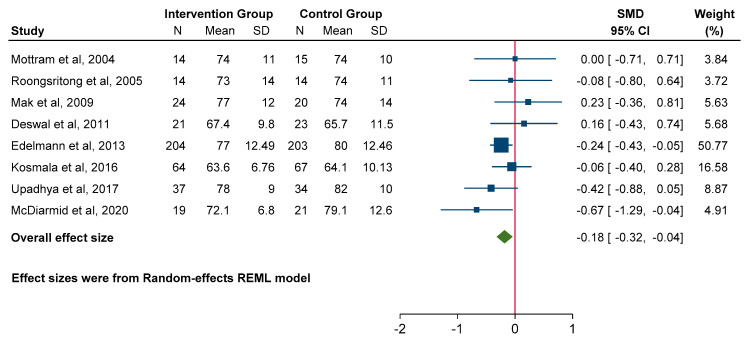
Forest plot for diastolic blood pressure.

A meta-analysis of eight studies revealed a clear benefit to MRA use for both SBP (SMD −0.27; 95% CI: −0.53 to −0.02, *p* = 0.03) and DBP (SMD −0.18; 95% CI: −0.32 to −0.04, *p* = 0.01) compared to the control group (Figure 7 and Figure 8).

### 3.6. Functional Parameters

**Figure 9:**
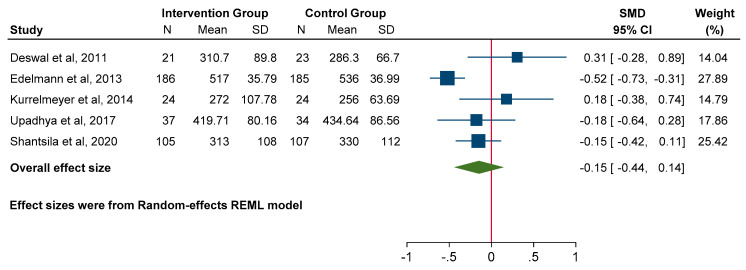
Forest plot for functional parameters: 6 min walk distance.

No significant difference was observed in 6MWD, with a moderate to high degree of heterogeneity noted (SMD −0.15; 95% CI: −0.44 to 0.14, *p* = 0.32; heterogeneity *p* = 0.01, I^2^ = 67%) (Figure 9). Two studies reported that MRA significantly improved peak VO_2_ [18,21], and three studies showed no significant effect [17,23,26]. Meta-analysis was not considered appropriate for this outcome, as quantitative data were present in only three studies and a substantial degree of heterogeneity was observed.

### 3.7. Quality-of-Life Measures

**Figure 10:**
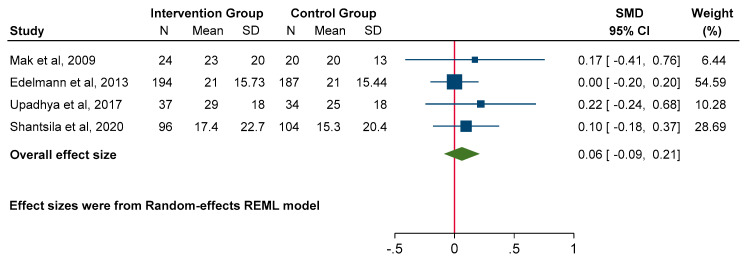
Forest plot for quality-of-life measures: Minnesota Living with Heart Failure Questionnaire.

Pooled data from three studies showed no significant effect of MRA on QoL using the MLWHFQ (SMD 0.06; 95% CI: −0.09 to 0.21, *p* = 0.42; heterogeneity *p* = 0.80, I^2^ = 0%), which was consistent with the descriptive data presented in the remaining studies not included in the meta-analysis (Figure 10). Given the small number of studies reporting KCCQ and variability in reporting of the NYHA functional class (considered as a categorical variable in this instance), it was deemed inappropriate to perform a meta-analysis for these outcomes. Descriptive data from these studies similarly reported no benefit with MRA [16,17,18,19,22,23,25].

A complete list of forest plots and funnel plots for primary and secondary outcomes can be found in Supplement S2.

### 3.8. Adverse Events

Hyperkalaemia was reported in 56 patients across five studies, of which three participants required hospitalisation [17,18,19,21,26]. Deterioration in renal function was the second most commonly reported adverse event in the MRA group, noted in three studies; however, these results did not reach statistical significance [17,18,21]. Breast pain in the MRA group was reported in three studies [14,18,26]. One study reported 6.8% of participants experiencing breast pain and 4.4% experiencing breast swelling in the MRA group [26]. Gynaecomastia was a rare complication noted in the MRA group in three participants across three studies [14,18,20]. There was one report of an allergic reaction to spironolactone, one report of syncope, and one report of hypotension [15,17].

## 4. Discussion

To our knowledge, this is the most updated and comprehensive meta-analysis that explores the benefits and harms of MRA use in patients with HFpEF. This review indicates that there is evidence that the use of MRAs in patients with HFpEF has the potential to improve diastolic function, with a significant difference noted in E/e’. In contrast to prior systematic reviews, our updated paper did not show statistically significant improvement in E/A ratio or LAVi [27,28]. According to the American Society of Echocardiography guidelines, E/e’ is a recommended parameter for the measurement of diastolic function due to its simplicity and accuracy, whereas other parameters may be confounded by the effect of age and other associated subclinical disorders [29]. Improvement in E/e’ is also more evident in studies with longer follow-up periods, which may suggest that the treatment time in the remaining included studies may be too short to demonstrate a noticeable benefit. Given that HFpEF is a chronic disease, a prolonged treatment time is warranted. Long-term studies are required to further validate the use of MRAs in clinical settings. We look forward to the results of the currently ongoing SPIRIT-HF trial, which aims to determine the effect of MRA use in HFmrEF (mid-range) and HFpEF with a follow-up period of 48 months [30].

Our findings are broadly consistent with previous meta-analyses evaluating the use of MRAs in HFpEF. For instance, Li et al. [27] and Kapelios et al. [28] reported improvements in certain echocardiographic parameters, such as E/e’ and LAVi, along with some functional benefits. However, these earlier reviews included fewer studies and did not incorporate more recent RCTs published after 2019. In contrast, our analysis synthesises data from 12 RCTs and reflects a broader and more contemporary patient cohort. Notably, while we confirmed a statistically significant improvement in E/e’ and blood pressure, we found no significant benefit in exercise capacity or quality of life. These findings refine and update the existing evidence, and underscore the need for more nuanced patient selection in future studies.

This meta-analysis demonstrates a statistically significant improvement in SBP and DBP with MRA use. While the exact mechanism is unclear, optimisation of BP is known to have beneficial effects on cardiac remodelling, and these results may explain the positive changes in E/e’ [26].

Improvement in parameters of diastolic function did not translate into clinical findings, with no significant improvement in 6MWD, peak VO_2_, or QoL parameters with MRA use. In fact, contradictory outcomes in 6MWD were noted in 2 studies [17,19]. This may be explained by the heterogeneous nature of HFpEF, which encompasses a range of phenotypes with differing pathophysiological drivers and responses to therapy. Subgroups such as patients with obesity, longstanding hypertension, or atrial fibrillation may exhibit varying degrees of exercise intolerance and QoL impairment that are influenced by factors beyond diastolic dysfunction [31]. For example, atrial fibrillation alters atrial compliance and filling pressures, while obesity can independently reduce functional capacity [31,32,33]. Most of the included RCTs did not stratify outcomes according to these clinical subtypes, limiting our ability to explore differential treatment effects. Future studies should focus on more precise phenotyping to tailor therapies more effectively in HFpEF.

Several adverse events have been reported with MRA use in HFpEF, the most significant of which is severe hyperkalaemia, leading to hospitalisation in a few cases [19]. Future use of MRAs would require specific inclusion criteria, strict dosing guidelines, and regular monitoring of potassium levels and renal function to ensure the risk-benefit ratio does not point toward harm.

The Treatment of Preserved Cardiac Function Heart Failure with Aldosterone Antagonist (TOPCAT) trial is the largest RCT to date that has investigated the effect of MRA on patients with HFpEF [27,28]. While the results of this trial were challenging to interpret due to substantial regional variations, subgroup analysis demonstrated a clinical benefit of MRA administration in select patients [34]. The TOPCAT trial used a spironolactone dose of 15–45 mg daily, while the majority of studies in this review used spironolactone 25 mg daily [34]. Further studies are required to determine whether an increased dose of MRA can mirror the TOPCAT results and lead to statistically significant beneficial effects; however, this must be balanced with side effects, as discussed.

The heterogeneous nature of HFpEF is increasingly understood through phenomapping studies, which use machine learning to classify patients into pathophysiologically distinct clusters [35]. These phenotypes—ranging from obese metabolic syndrome-predominant profiles to those dominated by atrial dysfunction or pulmonary hypertension—may exhibit markedly different responses to therapies, helping to explain the inconclusive pooled outcomes in functional and quality of life measures [35]. Incorporating phenomapping into future trial designs could help target interventions more effectively.

Current treatment options for HFpEF remain limited, with MRAs, SGLT2 inhibitors, and diuretics forming the mainstays of pharmacologic management. The high burden of comorbidities and frequent presence of atrial fibrillation further complicate treatment. Atrial fibrillation, in particular, is both a consequence and a contributor to diastolic dysfunction in HFpEF. Recent data suggest that catheter ablation may offer symptomatic and functional benefits in this population and warrants further investigation in randomised trials focused specifically on HFpEF cohorts [33].

### Study Limitations

Our meta-analysis has several limitations. According to the European Society of Cardiology 2021 guidelines, the definition of HFpEF requires an LVEF ≥ 50% [5]. Our review includes studies prior to 2021, with three study populations having an LVEF ≥ 45, which would include patients with both HFmrEF and HFpEF. Recent advances in echocardiography, such as the left atrial (LA) strain and global longitudinal strain (GLS), are increasingly recommended in the American Society of Echocardiography guidelines for assessing diastolic dysfunction. However, the majority of the RCTs included in our review were conducted before the widespread adoption of these techniques, so data on the LA strain and GLS were not available for our meta-analysis [29]. Moreover, multiple trials in this review included participants with concomitant use of RAAS inhibitors, and it is therefore difficult to determine whether the beneficial effect on BP and subsequent favourable effect on E/e’ can be attributed to MRA use alone. Assessment using 6MWD as a measure of functional capacity in patients with HFpEF is debatable, as HFpEF is prevalent in the elderly, who often have multiple comorbidities (such as arthritis and other mobility impairments) that preclude their optimal performance in 6MWD despite having adequate cardiac reserve [16]. In addition, several studies had a significant percentage of male participants (of >80% in some cases) [15,24], whereas HFpEF is a condition that is known to disproportionately affect women [18]. Small sample size, high drop-out rates, and subsequent low power are also limitations to consider. Risk of bias is unclear in many studies due to missing data that has not been adequately accounted for, lack of true randomisation, allocation concealment, and blinding. At the review level, it is to be noted that original investigators were contacted to supply missing data; however, a response was not received. Hence, meta-analysis was incomplete due to the lack of raw data available, which has the potential to influence and over-estimate the final results. However, we believe that considering the results of the descriptive data provided would have minimal impact on the overall conclusions of this review.

## 5. Conclusions

While there is evidence that MRA use in HFpEF can improve the echocardiographic parameters of diastolic function and BP control, this is not supported by improvements in functional capacity or QoL. Given that HFpEF is a chronic condition with significant morbidity and mortality with limited current guidelines or evidence-based therapy, clinicians who choose to use MRAs in these cohorts are recommended to use these medications cautiously. Close consideration of risk versus benefit at the individual level, with strict monitoring for adverse events, is advised. Further longer-term, appropriately randomised, adequately powered studies with accurate participant demographics are urgently needed to assess the effects of improving diastolic function and functional and clinical end points in this patient population.

## Figures and Tables

**Table 1:** Summary of the included studies evaluating MRAs in HFpEF.

Study (Year)	Country	Sample Size (MRA/Control)	Mean Age (Years)	LVEF (%)	Intervention	Duration	Key Outcomes Reported
Mottram (2004) [14]	Australia	15/15	62 ± 7	>50	Spironolactone 25 mg daily	6 months	Echo (E/A, EDT), BP
Roongsritong (2005) [15]	USA	15/15	~72	≥45	Spironolactone 25 mg daily	4 months	Echo (E/A, EDT), BNP, PICP
Mak (2009) [16]	Ireland	24/20	80 ± 7.8	>45	Eplerenone 25–50 mg daily	12 months	Echo, biomarkers, NYHA class, QoL
Deswal (2011) [17]	USA	23/23	~70	≥50	Eplerenone 25–50 mg daily	6.5 months	Echo, biomarkers, 6MWD, QoL
Edelmann (2013) [18]	Germany, Austria	213/209	67 ± 8	≥50	Spironolactone 25 mg daily	12 months	Echo, biomarkers, 6MWD, peak VO_2_, QoL
Kurrelmeyer (2014) [19]	USA	24/24	70	≥50	Spironolactone 25 mg daily	6 months	Echo, biomarkers, 6MWD, QoL
Shah (2015) [20]	USA, Russia, Georgia	121/118	~69	≥45	Spironolactone 15–45 mg daily	18 months	Echo
Kosmala (2016) [21]	Australia	75/75	67 ± 9	>50	Spironolactone 25 mg daily	6 months	Echo, biomarkers, peak VO_2_
Kosmala (2019) [22]	Australia	51/54	64 ± 8	>50	Spironolactone 25 mg daily	6 months	Echo, biomarkers, peak VO_2_
Upadhya (2017) [23]	USA	42/38	71 ± 1	≥50	Spironolactone 25 mg daily	9 months	Echo, CMR, biomarkers, 6MWD, QoL
McDiarmid (2020) [24]	UK	27/24	75 ± 7.3	>50	Spironolactone 25 mg daily	6 months	Echo, CMR, biomarkers
Shantsila (2020) [25]	UK	125/125	72.3 ± 7.4	≥55	Spironolactone 25 mg daily	2 years	Echo, biomarkers, 6MWD, QoL

Abbreviations: MRA = mineralocorticoid receptor antagonist; Echo = echocardiography; BP = blood pressure; QoL = quality of life; 6MWD = 6 min walk distance; CMR = cardiac magnetic resonance; BNP = natriuretic peptides; PICP = serum biomarkers.

## Supplementary Materials

The following supporting information can be downloaded at https://www.mdpi.com/article/10.3390/jcm14103598/s1, Supplement S1: Search terms, Search databases, Supplement S2: Forest plots and funnel plots for primary and secondary outcomes, Table S1: Characteristics of included studies, Table S2: Risk of bias assessment Joanna Briggs Institute of Critical Appraisal Tools Checklist for RCTs.

## Abbreviations

The following abbreviations are used in this manuscript:

HFpEF	Heart failure with preserved ejection fraction
MRA	Mineralocorticoid receptor antagonists
RCT	Randomised controlled trials
NYHA	New York Heart Association
LVEF	Left ventricular ejection fraction
SBP	Systolic blood pressure
DBP	Diastolic blood pressure
6MWD	6 min walk distance
MLWHFQ	Minnesota living with heart failure questionnaire
KCCQ	Kansas City cardiomyopathy questionnaire
QoL	Quality of life

## References

[1] Pieske B., Tschope C., de Boer R., Fraser A.G., Anker S.D., Donal E., Edelmann F., Fu M., Guazzi M., Lam C.S.P. (2019). How to diagnose heart failure with preserved ejection fraction: The HFA-PEFF diagnostic algorithm: A consensus recommendation from the Heart Failure Association (HFA) of the European Society of Cardiology (ESC). Eur. Hear. J..

[2] Pitt B., Zannand F., Remme W.J., Cody R., Castaigne A., Perez A., Palensky J., Wittes J. (1999). The Effect of Spironolactone on Morbidity and Mortality in Patients with Severe Heart Failure. N. Engl. J. Med..

[3] Anker S.D., Filippatos B.G., Ferreira J.P., Ferreira J.P., Bocchi E., Böhm M., Brunner–La Rocca H.-P., Choi D.-J., Chopra V., Chuquiure-Valenzuela E. (2021). Empagliflozin in Heart Failure with a Preserved Ejection Fraction. N. Engl. J. Med..

[4] Nassif M.E., Windsor S.L., Borlaug B.A., Kitzman D.W., Shah S.J., Tang F., Khariton Y., Malik A.O., Khumri T., Umpierrez G. (2021). The SGLT2 inhibitor dapagliflozin in heart failure with preserved ejection fraction: A multicenter randomized trial. Nat. Med..

[5] McDonagh T.A., Meta M., Adamo M., Gardner R.S., Baumbach A., Böhm M., Burri H., Butler J., Čelutkienė J., Chioncel O. (2023). 2023 Focused Update of the 2021 ESC Guidelines for the diagnosis and treatment of acute and chronic heart failure: Developed by the task force for the diagnosis and treatment of acute and chronic heart failure of the European Society of Cardiology (ESC) With the special contribution of the Heart Failure Association (HFA) of the ESC. Eur. Heart J..

[6] Vizzardi E., Regazzoni V., Caretta G., Gavazzoni M., Sciatti E., Bonadei I., Trichaki E., Raddino R., Metra M. (2014). Mineralocorticoid receptor antagonist in heart failure: Past, present and future perspectives. Int. J. Cardiol. Heart Vessel..

[7] Brown N.J. (2003). Eplerenone: Cardiovascular Protection. Circulation.

[8] Cittadini A., Monti M.G., Isgaard J., Casaburi C., Strömer H., Di Gianni A., Serpico R., Saldamarco L., Vanasia M., Saccà L. (2003). Aldosterone receptor blockade improves left ventricular remodeling and increases ventricular fibrillation threshold in experimental heart failure. Cardiovasc. Res..

[9] Bakris G.L., Agarwal R., Anker S.D., Pitt B., Ruilope L.M., Rossing P., Kolkhof P., Nowack C., Schloemer P., Joseph A. (2020). Effect of Finerenone on Chronic Kidney Disease Outcomes in Type 2 Diabetes. N. Engl. J. Med..

[10] Pitt B., Filippatos G., Agarwal R., Anker S.D., Bakris G.L., Rossing P., Joseph A., Kolkhof P., Nowack C., Schloemer P. (2021). Cardiovascular Events with Finerenone in Kidney Disease and Type 2 Diabetes. N. Engl. J. Med..

[11] Page M.J., McKenzie J.E., Bossuyt P.M., Boutron I., Hoffmann T.C., Mulrow C.D., Shamseer L., Tetzlaff J.M., Akl E.A., Brennan S.E. (2021). The PRISMA 2020 statement: An updated guideline for reporting systematic reviews. BMJ.

[12] (2021). Jbi.global. https://jbi.global/critical-appraisal-tools.

[13] Higgins J.P.T., Thomas J., Chandler J., Cumpston M., Li T., Page M.J., Welch V.A. (2021). Cochrane Handbook for Systematic Reviews of Interventions Version 6.2 (Updated February 2021). https://www.training.cochrane.org/handbook.

[14] Mottram P.M., Haluska B., Leano R., Cowley D., Stowasser M., Marwick T.H. (2004). Effect of aldosterone antagonism on myocardial dysfunction in hypertensive patients with diastolic heart failure. Circulation.

[15] Roongsritong C., Sutthiwan P., Bradley J., Simoni J., Power S., Meyerrose G.E. (2005). Spironolactone improves diastolic function in the elderly. Clin. Cardiol..

[16] Mak G.J., Ledwidge M.T., Watson C.J., Phelan D.M., Dawkins I.R., Murphy N.F., Patle A.K., Baugh J.A., McDonald K.M. (2009). Natural history of markers of collagen turnover in patients with early diastolic dysfunction and impact of eplerenone. J. Am. Coll. Cardiol..

[17] Deswal A., Richardson P., Bozkurt B., Mann D.L. (2011). Results of the Randomized Aldosterone Antagonism in Heart Failure with Preserved Ejection Fraction trial (RAAM-PEF). J. Card. Fail..

[18] Edelmann F., Wachter R., Schmidt A.G., Kraigher-Krainer E., Colantonio C., Kamke W., Duvinage A., Stahrenberg R., Durstewitz K., Löffler M. (2013). Effect of spironolactone on diastolic function and exercise capacity in patients with heart failure with preserved ejection fraction: The Aldo-DHF randomized controlled trial. JAMA.

[19] Kurrelmeyer K.M., Ashton Y., Xu J., Nagueh S.F., Torre-Amione G., Deswal A. (2014). Effects of spironolactone treatment in elderly women with heart failure and preserved left ventricular ejection fraction. J. Card. Fail..

[20] Shah A.M., Claggett B., Sweitzer N.K., Shah S.J., Deswal A., Anand I.S., Fleg J.L., Pitt B., Pfeffer M.A., Solomon S.D. (2015). Prognostic Importance of Changes in Cardiac Structure and Function in Heart Failure With Preserved Ejection Fraction and the Impact of Spironolactone. Circ. Heart Fail..

[21] Kosmala W., Rojek A., Przewlocka-Kosmala M., Wright L., Mysiak A., Marwick T.H. (2016). Effect of Aldosterone Antagonism on Exercise Tolerance in Heart Failure With Preserved Ejection Fraction. J. Am. Coll. Cardiol..

[22] Kosmala W., Przewlocka-Kosmala M., Marwick T.H. (2019). Association of Active and Passive Components of LV Diastolic Filling With Exercise Intolerance in Heart Failure With Preserved Ejection Fraction: Mechanistic Insights From Spironolactone Response. JACC Cardiovasc. Imaging.

[23] Upadhya B., Hundley W.G., Brubaker P.H., Morgan T.M., Stewart K.P., Kitzman D.W. (2017). Effect of Spironolactone on Exercise Tolerance and Arterial Function in Older Adults with Heart Failure with Preserved Ejection Fraction. J. Am. Geriatr. Soc..

[24] McDiarmid A.K., Swoboda P.P., Erhayiem B., Bounford K.A., Bijsterveld P., Tyndall K., Fent G.J., Garg P., Dobson L.E., Musa T.A. (2020). Myocardial Effects of Aldosterone Antagonism in Heart Failure With Preserved Ejection Fraction. J. Am. Heart Assoc..

[25] Shantsila E., Shahid F., Sun Y., Deeks J., Calvert M., Fisher J.P., Kirchhof P., Gill P.S., Lip G.Y.H. (2020). Spironolactone in Atrial Fibrillation With Preserved Cardiac Fraction: The IMPRESS-AF Trial. J. Am. Heart Assoc..

[26] Pitt B., Pfeffer M.A., Assmann S.F., Boineau R., Anand I.S., Claggett B., Clausell N., Desai A.S., Diaz R., Fleg J.L. (2014). Spironolactone for heart failure with preserved ejection fraction. N. Engl. J. Med..

[27] Li S., Zhang X., Dong M., Gong S., Shang Z., Jia X., Chen W., Yang J., Li J. (2018). Effects of spironolactone in heart failure with preserved ejection fraction: A meta-analysis of randomized controlled trials. Medicine.

[28] Kapelios C.J., Murrow J.R., Nührenberg T.G., Montoro Lopez M.N. (2019). Effect of mineralocorticoid receptor antagonists on cardiac function in patients with heart failure and preserved ejection fraction: A systematic review and meta-analysis of randomized controlled trials. Heart Fail. Rev..

[29] Nagueh S.F., Smiseth O.A., Appleton C.P., Byrd B.F., Dokainish H., Edvardsen T., Flachskampf F.A., Gillebert T.C., Klein A.L., Lancellotti P. (2016). Recommendations for the evaluation of left ventricular diastolic function by echocardiography: An update from the American Society of Echocardiography and the European Association of Cardiovascular Imaging. J. Am. Soc. Echocardiogr..

[30] US National Library of Medicine (2021). Spironolactone in the Treatment of Heart Failure (SPIRIT-HF). https://clinicaltrials.gov/ct2/show/NCT04727073.

[31] van Dalen B.M., Chin J.F., Motiram P.A., Hendrix A., Emans M.E., Brugts J.J., Westenbrink B.D., de Boer R.A. (2025). Challenges in the diagnosis of heart failure with preserved ejection fraction in individuals with obesity. Cardiovasc. Diabetol..

[32] Kittipibul V., Lam C.S.P. (2025). Heart failure with preserved ejection fraction and atrial fibrillation: Epidemiology, pathophysiology, and diagnosis interplay. Heart Fail. Rev..

[33] La Fazia V.M., Pierucci N., Mohanty S., Chiricolo G., Natale A. (2025). Atrial fibrillation ablation in heart failure with preserved ejection fraction. Card. Electrophysiol. Clin..

[34] Pfeffer M., Claggett B., Assmann S.F., Boineau R., Anand I.S., Clausell N., Desai A.S., Diaz R., Fleg J.L., Gordeev I. (2015). Regional Variation in Patients and Outcomes in the Treatment of Preserved Cardiac Function Heart Failure With an Aldosterone Antagonist (TOPCAT) Trial. Circulation.

[35] Peters A.E., Tromp J., Shah S.J., Lam C.S., Lewis G.D., Borlaug B.A., Sharma K., Pandey A., Sweitzer N.K., Kitzman D.W. (2022). Phenomapping in heart failure with preserved ejection fraction: Insights, limitations, and future directions. Cardiovasc. Res..

