# Supplementary material for: Mineralocorticoid Receptor Antagonists in Heart Failure with Preserved Ejection Fraction: A Systematic Review and Meta-Analysis
Source: J Clin Med. 2025 May 21;14(10):3598. doi: 10.3390/jcm14103598 (PMC12112577; doi:10.3390/jcm14103598)
Supplement: Supplementary file 1 [file jcm-14-03598-s001.zip › Supplement S1 - search terms .pdf]

Supplement S1

Search terms

- Concept 1:
    - "Heart failure, diastolic" OR "diastolic heart failure" OR "heart failure with normal ejection fraction" OR "heart failure with preserved ejection fraction" OR "HFpEF" OR "diastolic dysfunction"
- Concept 2:
    - "Mineralocorticoid receptor antagonist" OR "mineralocorticoid receptor blocker" OR "aldosterone antagonist" OR "aldosterone blockade" OR "spironolactone" OR "eplerenone"

Search databases:

- CINAHL, Ovid Medline, EMBASE, Cochrane Central register of Controlled Trials (CENTRAL), Scopus, Science direct, ProQuest, Google Scholar (for manual searching of reference list)
