# Supplementary material for: Mineralocorticoid Receptor Antagonists in Heart Failure with Preserved Ejection Fraction: A Systematic Review and Meta-Analysis
Source: J Clin Med. 2025 May 21;14(10):3598. doi: 10.3390/jcm14103598 (PMC12112577; doi:10.3390/jcm14103598)

Supplement S2: Forest plots and funnel plots for primary and secondary outcomes.

3.4 Diastolic function

<u>Early mitral inflow velocity (E/e')</u>

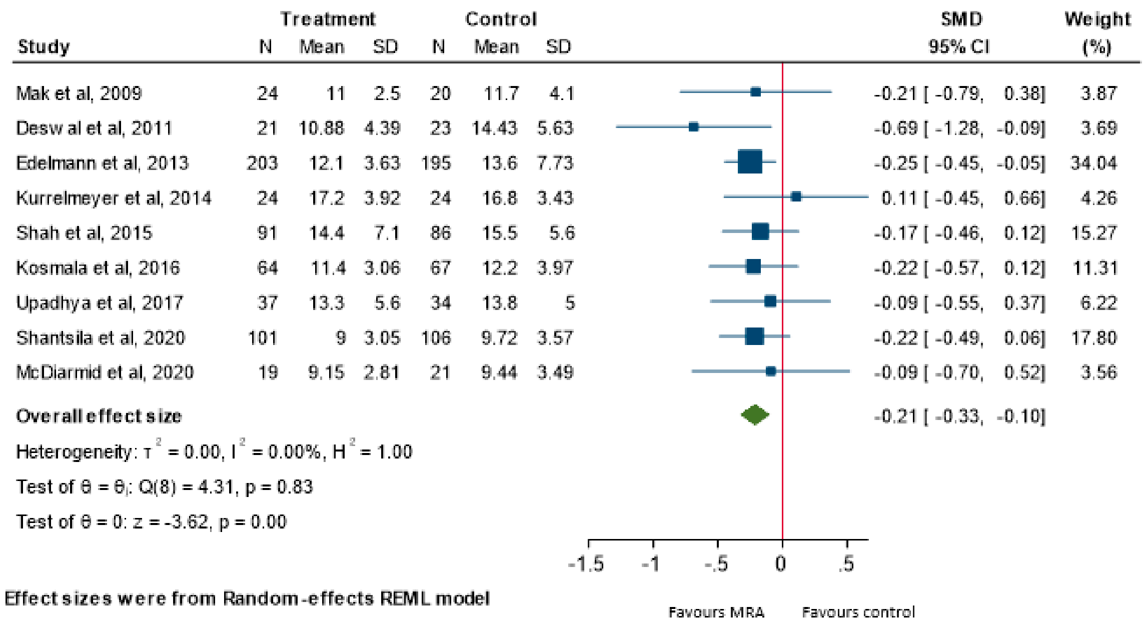

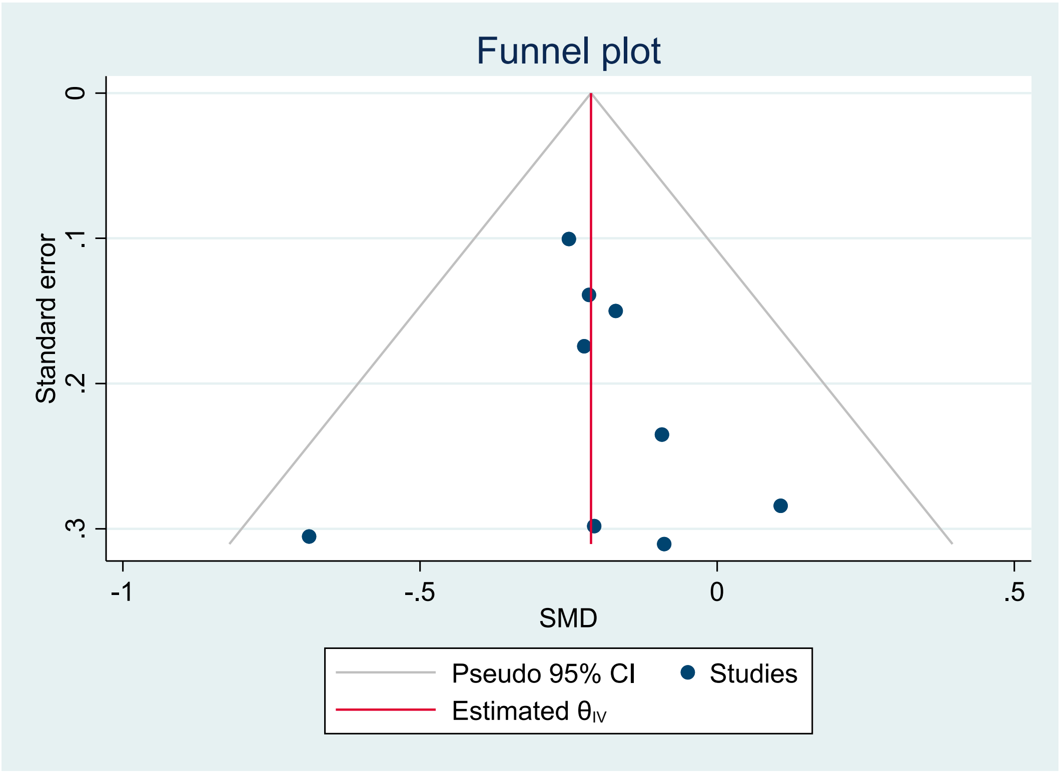

Mitral annular early diastolic velocity ratio (E/A ratio)

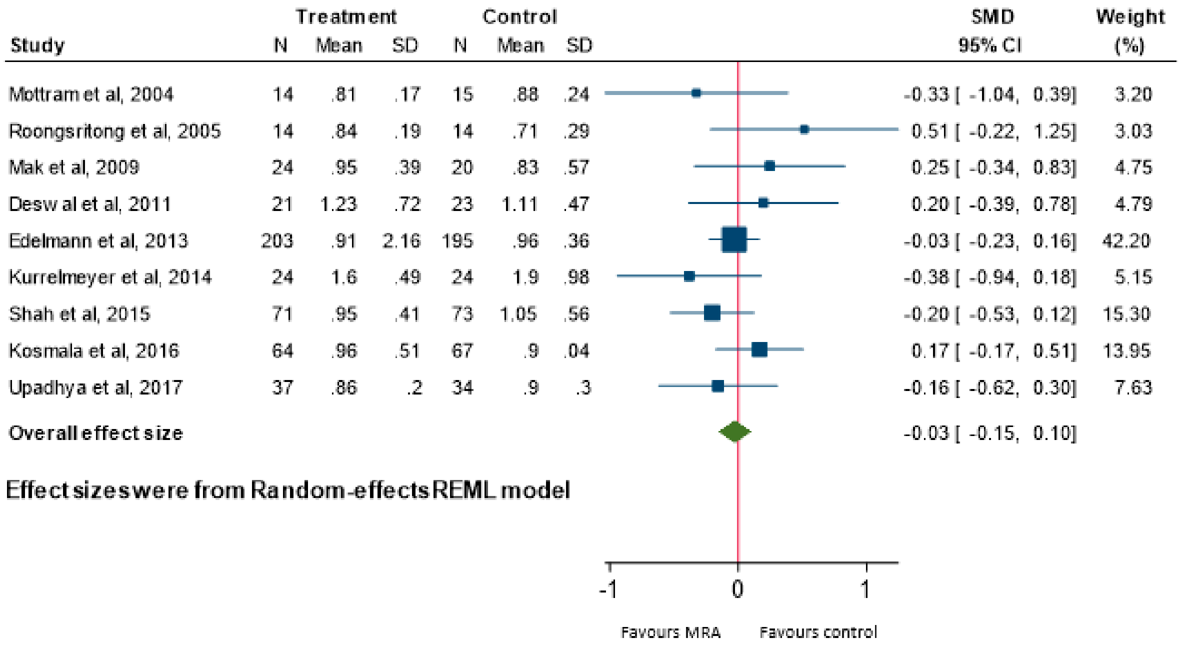

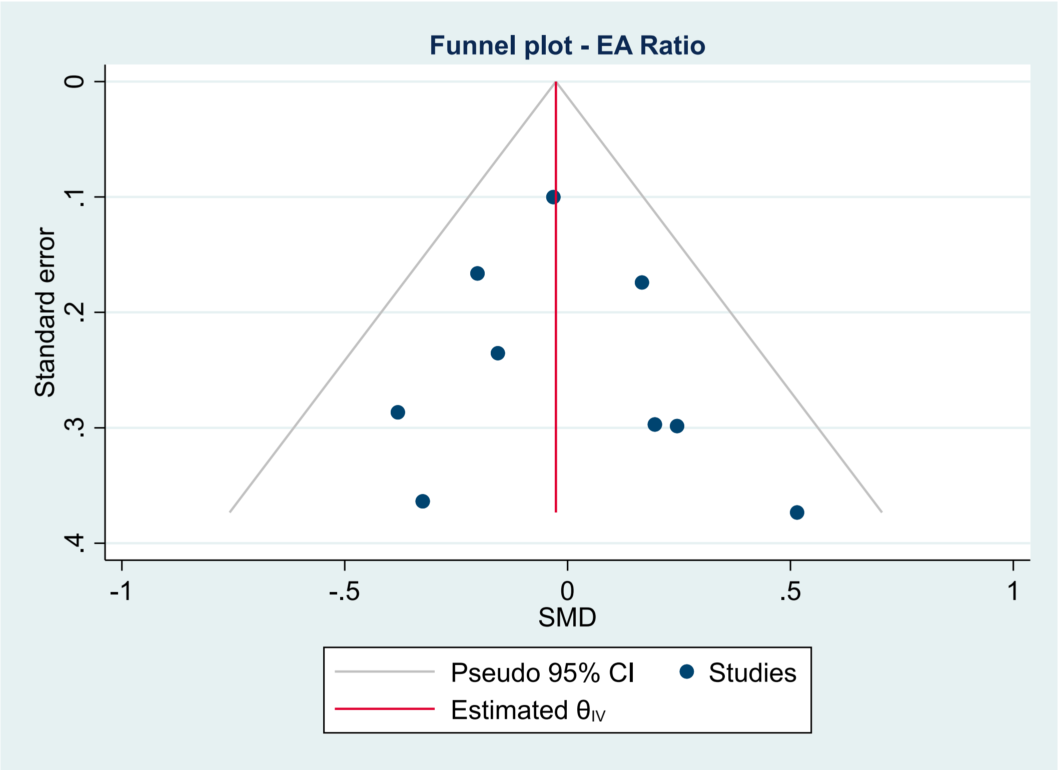

Left atrial volume index (LAVi)

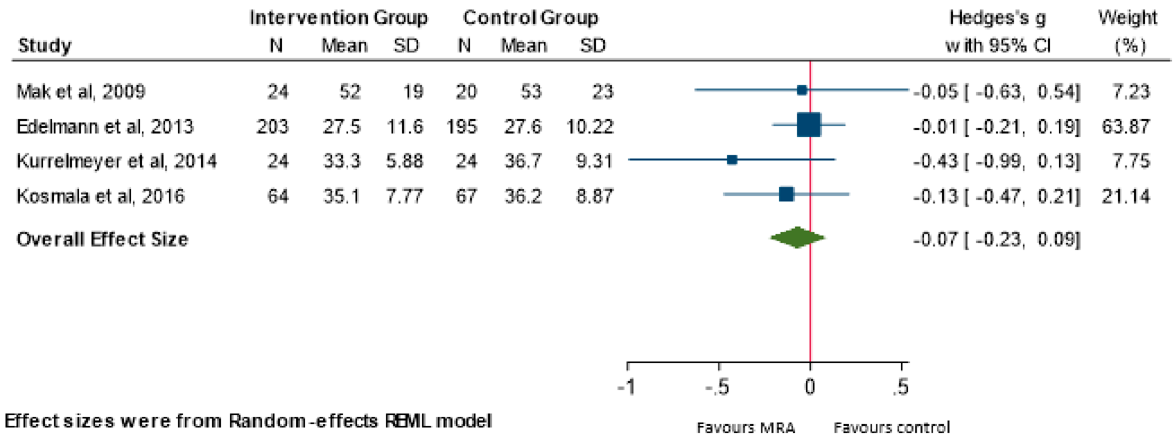

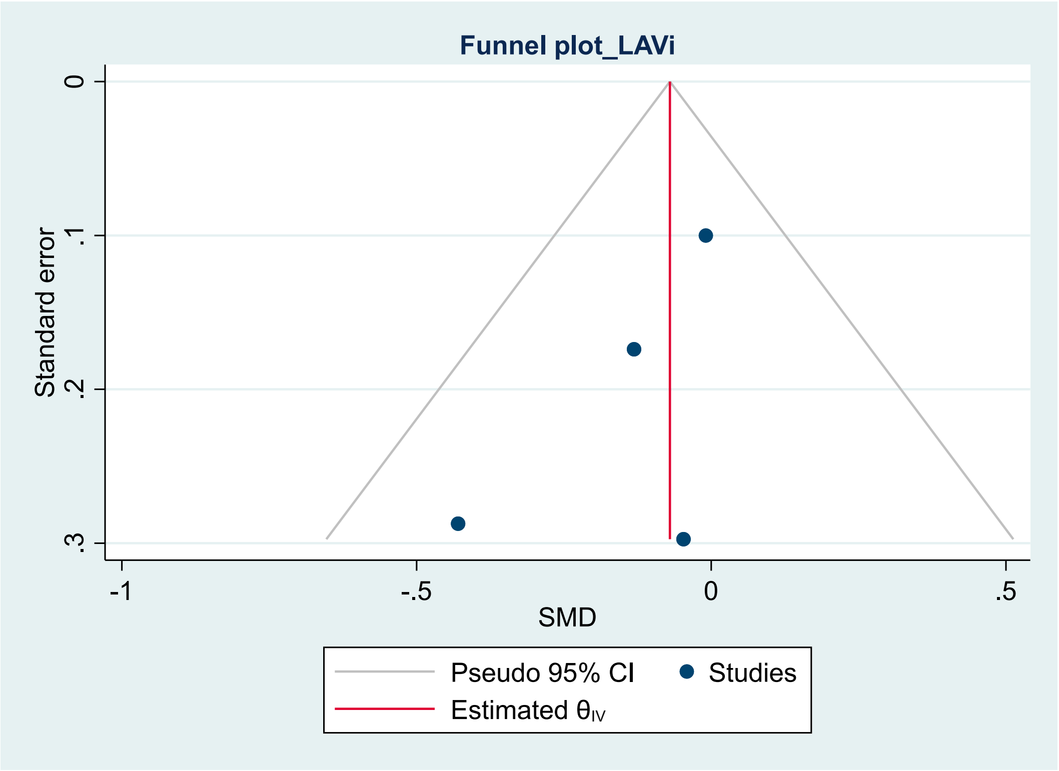

Deceleration time (DT)

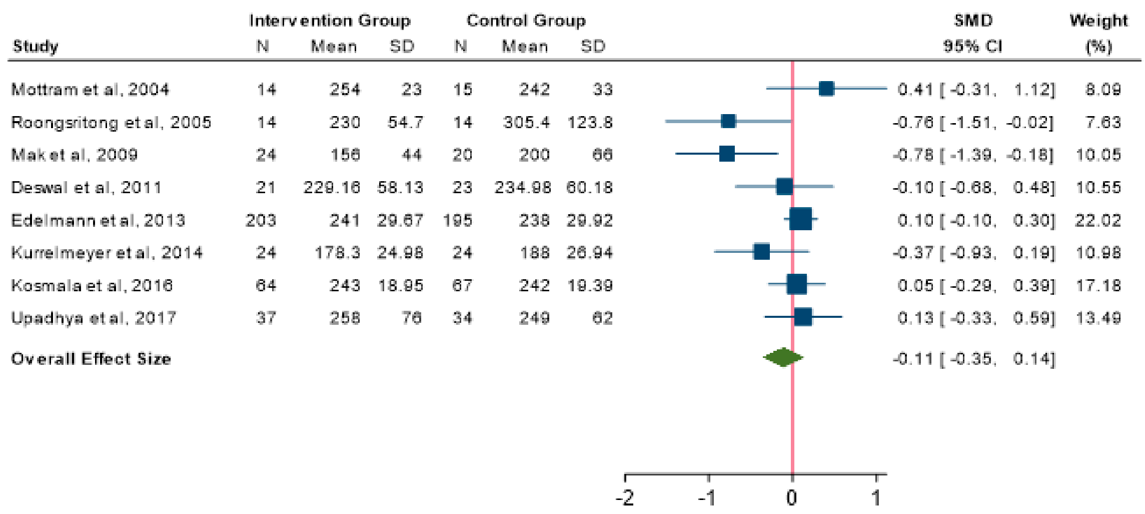

Study	Intervention Group N	Intervention Group Mean	Intervention Group SD	Control Group N	Control Group Mean	Control Group SD	SMD 95% CI	Weight (%)
Mottram et al, 2004	14	254	23	15	242	33	0.41 [-0.31, 1.12]	8.09
Roongsritong et al, 2005	14	230	54.7	14	305.4	123.8	-0.76 [-1.51, -0.02]	7.63
Mak et al, 2009	24	156	44	20	200	66	-0.78 [-1.39, -0.18]	10.05
Deswal et al, 2011	21	229.16	58.13	23	234.98	60.18	-0.10 [-0.68, 0.48]	10.55
Edelmann et al, 2013	203	241	29.67	195	238	29.92	0.10 [-0.10, 0.30]	22.02
Kurrelmeyer et al, 2014	24	178.3	24.98	24	188	26.94	-0.37 [-0.93, 0.19]	10.98
Kosmala et al, 2016	64	243	18.95	67	242	19.39	0.05 [-0.29, 0.39]	17.18
Upadhya et al, 2017	37	258	76	34	249	62	0.13 [-0.33, 0.59]	13.49
Overall Effect Size							-0.11 [-0.35, 0.14]	

Effect Sizes were from Random-effects REML model

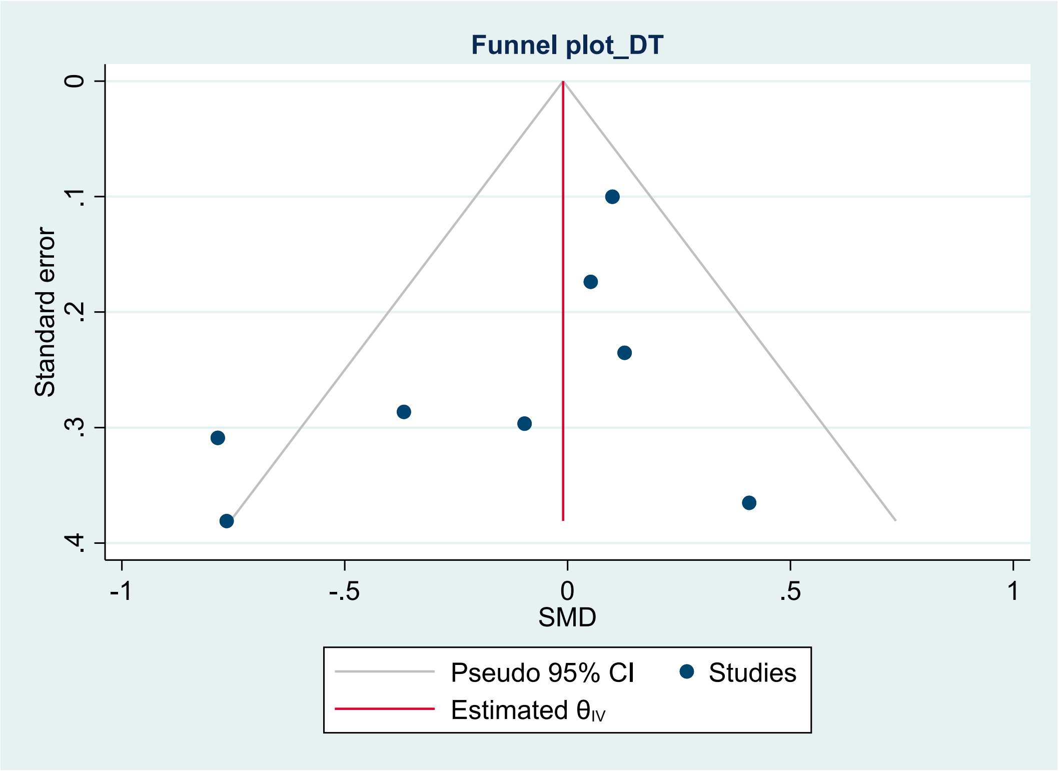

Left ventricular mass index (LVMi)

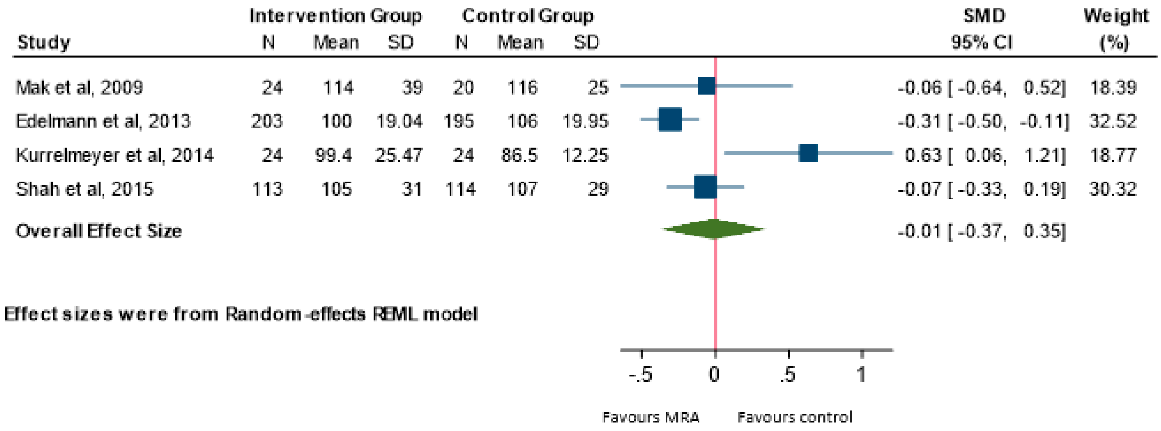

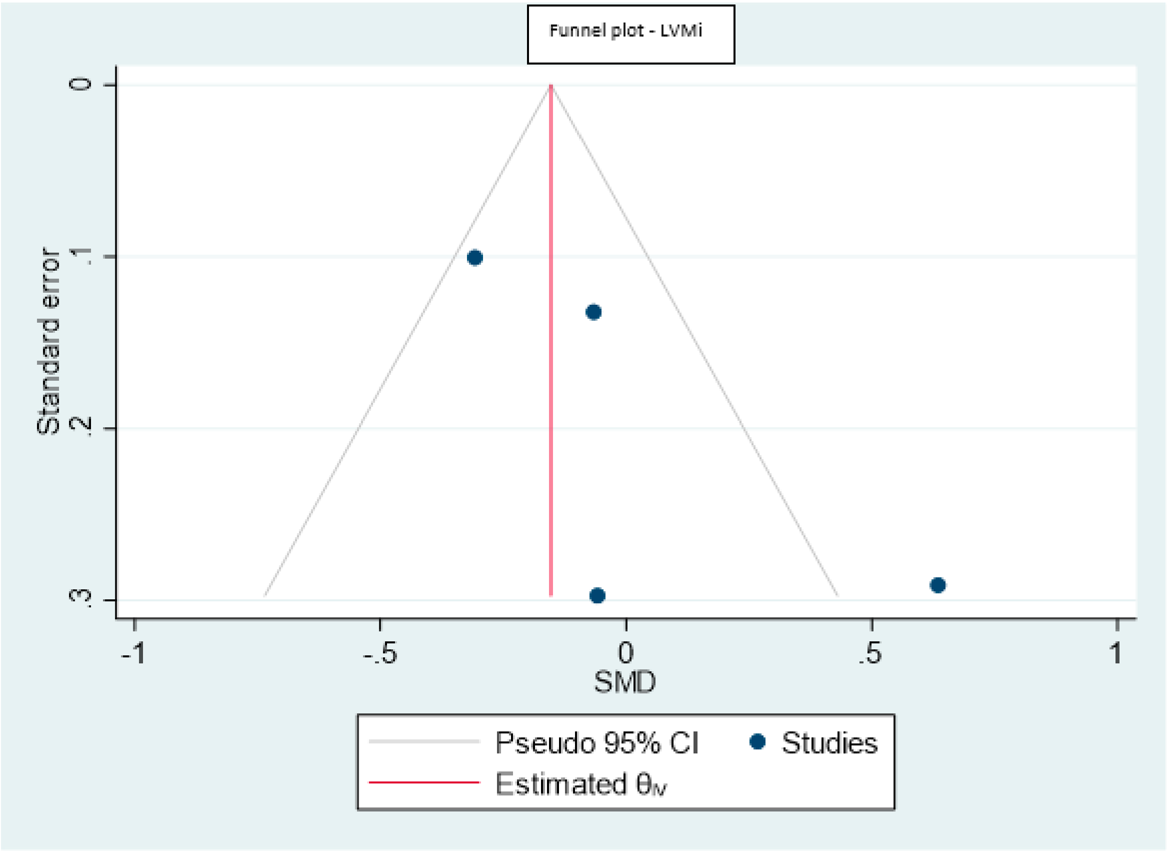

3.5 Effect on blood pressure

Systolic blood pressure (SBP)

Study	Intervention Group N	Intervention Group Mean	Intervention Group SD	Control Group N	Control Group Mean	Control Group SD	SMD 95% CI	Weight (%)
Mottram et al, 2004	14	140	14	15	137	19	0.17 [-0.54, 0.88]	8.36
Roongsritong et al, 2005	14	138	15	14	144	20	-0.33 [-1.05, 0.39]	8.13
Mak et al, 2009	24	145	22	20	141	21	0.18 [-0.40, 0.77]	10.66
Deswal et al, 2011	21	126.4	15.8	23	130.2	18.7	-0.21 [-0.80, 0.37]	10.69
Edelmann et al, 2013	204	128	14.43	196	137	14.14	-0.63 [-0.83, -0.43]	21.70
Kosmala et al, 2016	64	162.7	13.16	67	163	12.81	-0.02 [-0.36, 0.32]	17.21
Upadhya et al, 2017	37	176	22	34	183	23	-0.31 [-0.77, 0.16]	13.55
McDiarmid et al, 2020	19	120.2	13.5	21	129.6	9.9	-0.78 [-1.42, -0.15]	9.70
Overall effect size							-0.27 [-0.53, -0.02]	

Effect sizes were from Random-effects REML model

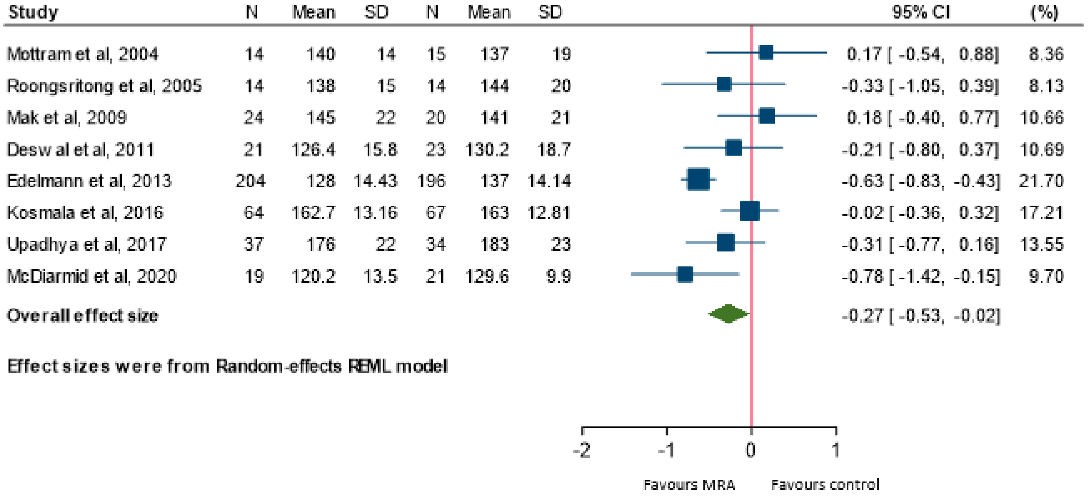

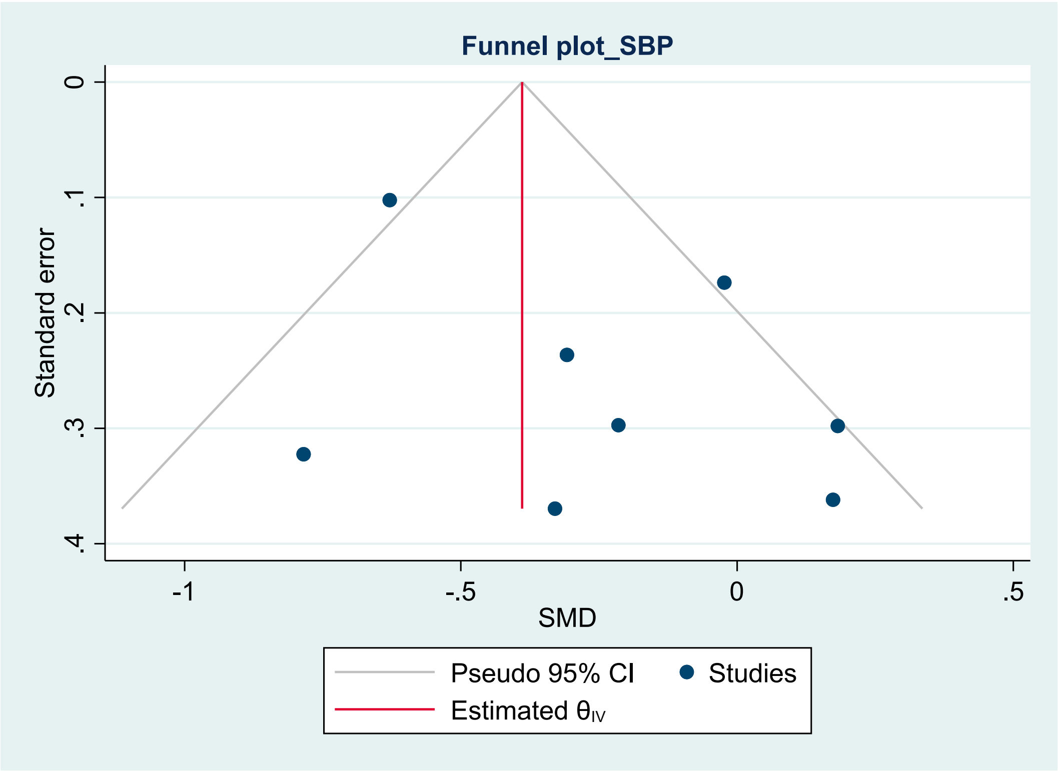

Diastolic blood pressure (BP)

Study	Intervention Group N	Intervention Group Mean	Intervention Group SD	Control Group N	Control Group Mean	Control Group SD	SMD 95% CI	Weight (%)
Mottram et al, 2004	14	74	11	15	74	10	0.00 [-0.71, 0.71]	3.84
Roongsritong et al, 2005	14	73	14	14	74	11	-0.08 [-0.80, 0.64]	3.72
Mak et al, 2009	24	77	12	20	74	14	0.23 [-0.36, 0.81]	5.63
Deswal et al, 2011	21	67.4	9.8	23	65.7	11.5	0.16 [-0.43, 0.74]	5.68
Edelmann et al, 2013	204	77	12.49	203	80	12.46	-0.24 [-0.43, -0.05]	50.77
Kosmala et al, 2016	64	63.6	6.76	67	64.1	10.13	-0.06 [-0.40, 0.28]	16.58
Upadhya et al, 2017	37	78	9	34	82	10	-0.42 [-0.88, 0.05]	8.87
McDiarmid et al, 2020	19	72.1	6.8	21	79.1	12.6	-0.67 [-1.29, -0.04]	4.91
Overall effect size							-0.18 [-0.32, -0.04]	

Effect sizes were from Random-effects REML model

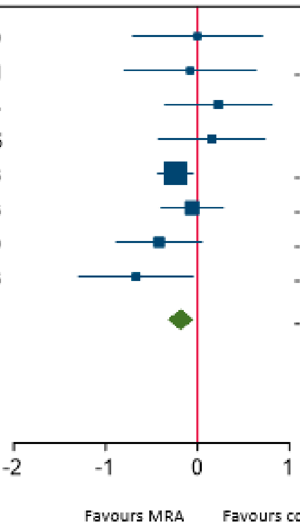

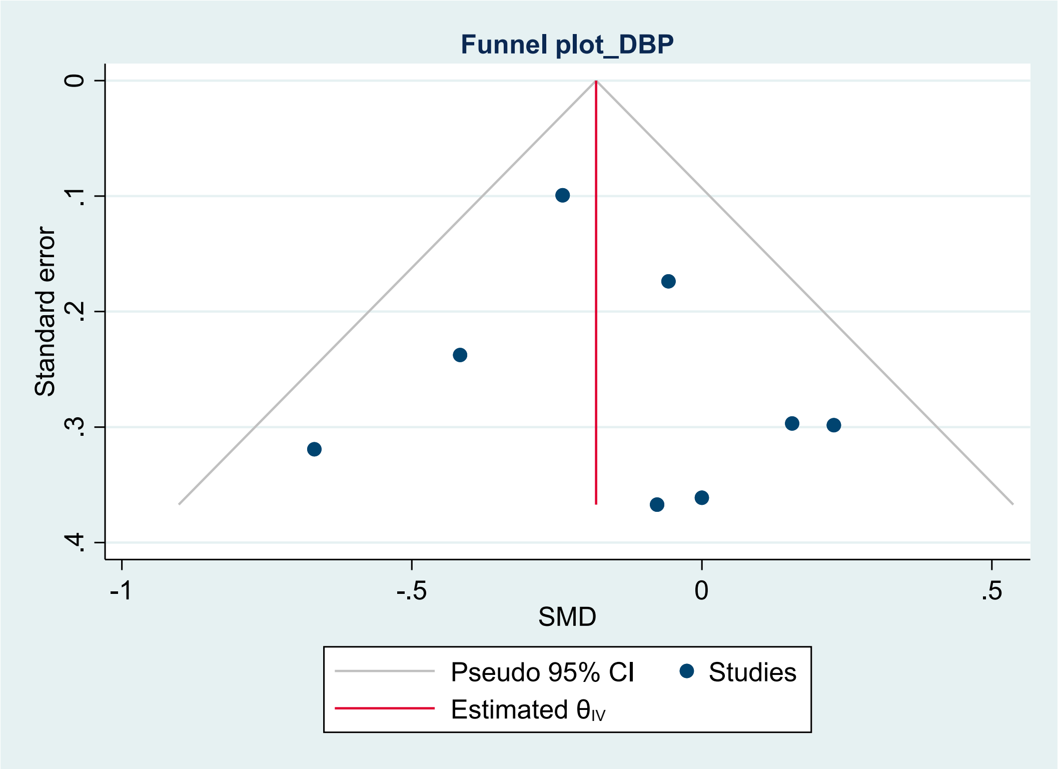

3.6 Functional parameters

<u>6-minute walk distance (6MWD)</u>

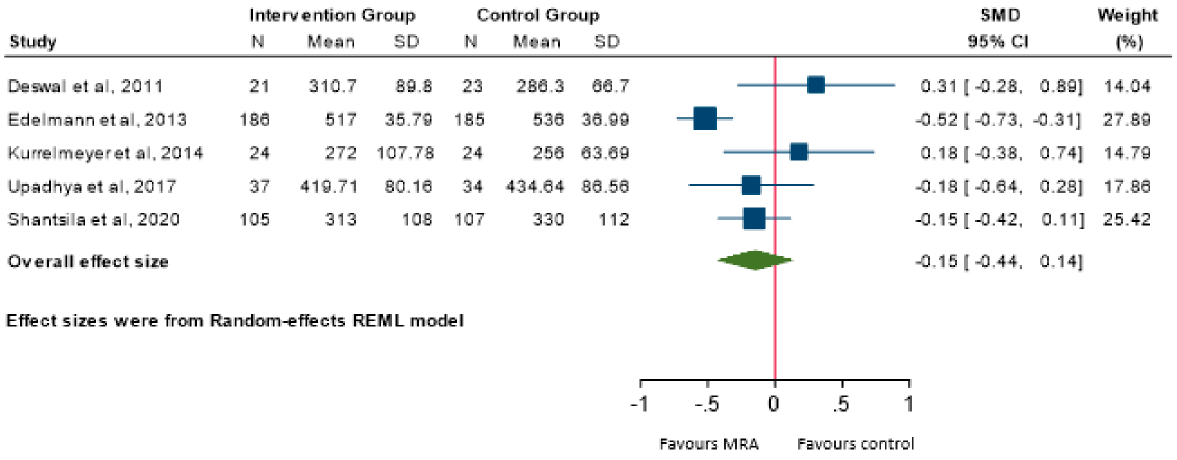

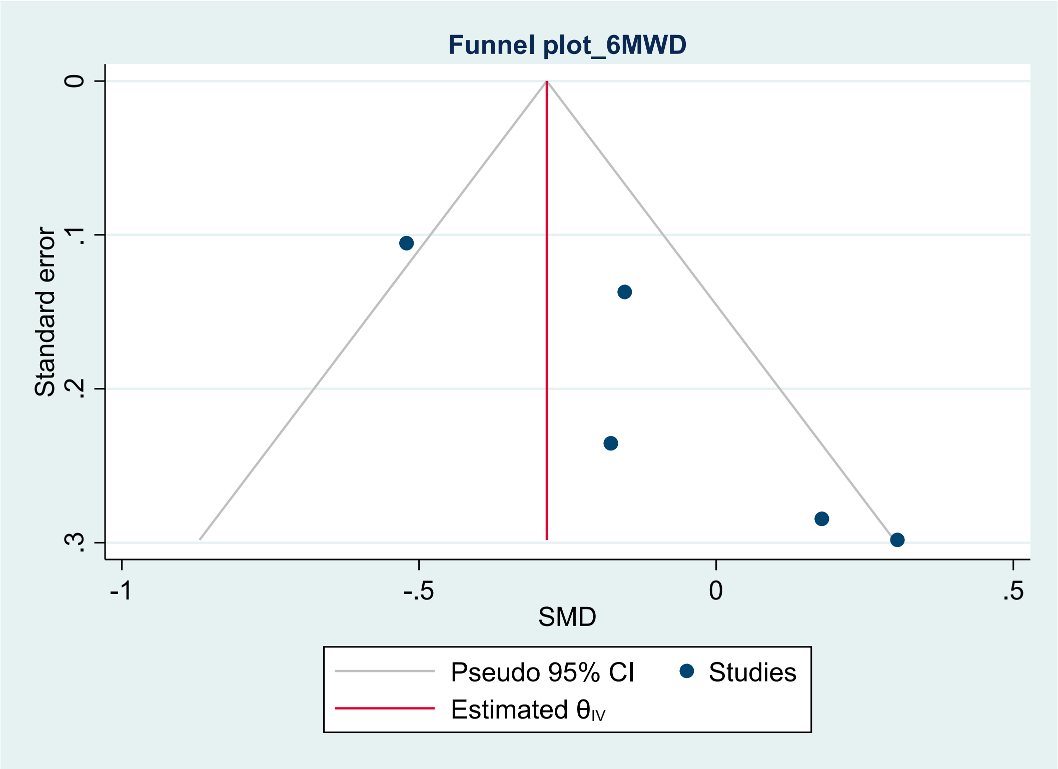

3.7 Quality of life measures

Minnesota Living With Heart Failure Questionnaire (MLWHFQ)

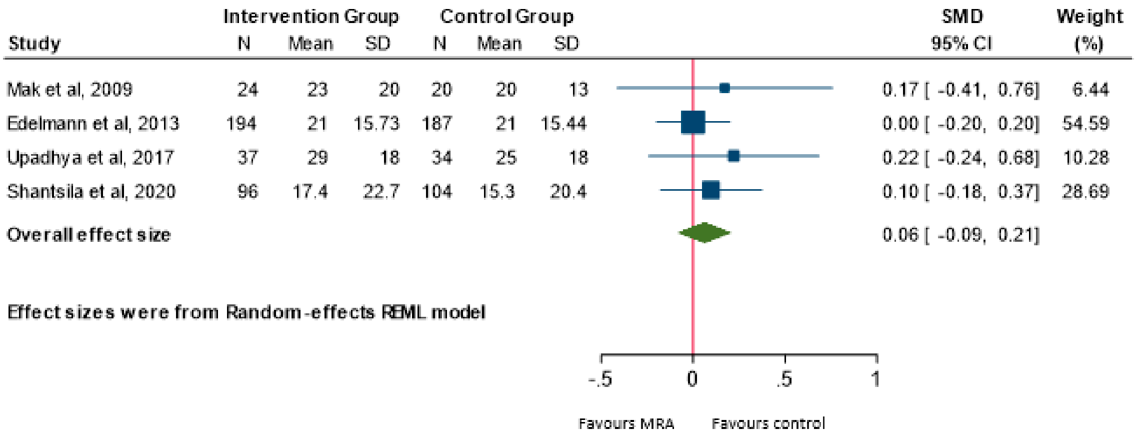

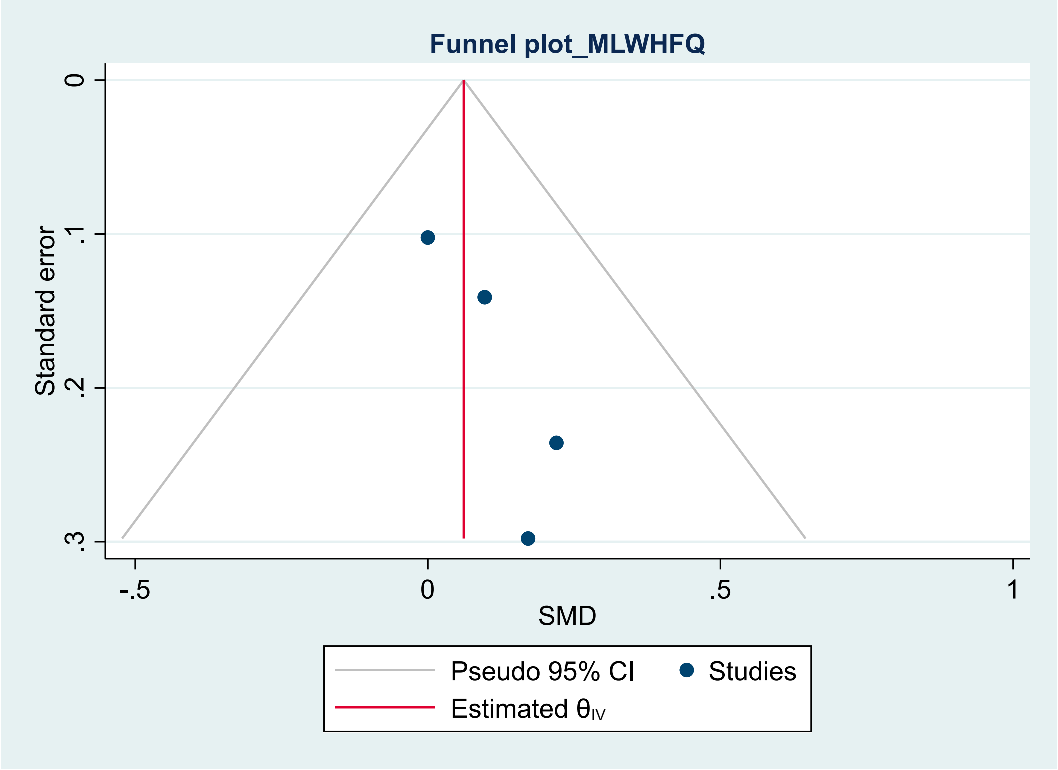

Supplement: Supplementary file 1 [file jcm-14-03598-s001.zip › Supplement S2 forest plots and funnel plots.pdf]
