# Supplementary material for: Mineralocorticoid Receptor Antagonists in Heart Failure with Preserved Ejection Fraction: A Systematic Review and Meta-Analysis
Source: J Clin Med. 2025 May 21;14(10):3598. doi: 10.3390/jcm14103598 (PMC12112577; doi:10.3390/jcm14103598)
Supplement: Supplementary file 1 [file jcm-14-03598-s001.zip › Table S1 - baseline characteristics.pdf]

Table S1: Characteristics of included studies

Study (year)	Country	Participant features	Mean age (years)	LVEF (%)	MRA (n)	Control (n)	Intervention	Control	Follow up duration	BP measures	Echo parameters	Serum biomarkers	Functional parameters	Quality of life measures
**Mottram (2004)**	Australia	Overweight (BMI 31±5 kg/m^2)	62±7	>50	15	15	Spironolactone 25mg daily	Placebo	6 months	SBP, DBP	E/A, EDT, long axis SR, peak systolic strain	NR	NR	NR
**Roongsritong (2005)**	United States	Elderly (age 60 – 85 years)	71.0±5.5 (MRA)<br>72.1±6.9 (control)	≥45	15	15	Spironolactone 25mg daily	Placebo	4 months	SBP, DBP	E/A, EDT	BNP, PICP	NR	NR
**Mak (2009)**	Ireland	NYHA class II – IV	80±7.8	>45	24	20	Eplerenone 25mg daily from 0 – 6 months<br>Eplerenone 50mg daily from 6 – 12 months	Standard therapy	6 and 12 months	SBP, DBP	E/A, EDT, E/e’, LVEF, LAVi, LVMi	PICP, PINP, PIIINP, CITP, MMP-2	NYHA class	Minnesota Living with Heart Failure Questionnaire
**Deswal (2011)**	United States	NYHA class II – III	72.2±9.8 (MRA)	≥50	23	23	Eplerenone 25mg daily from 0 – 2 weeks	Placebo	6.5 months	SBP, DBP	E/A, EDT, E/e’	BNP, PICP, PINP, PIIINP, CITP	6MWD, NYHA class	Kansas City Cardiomyopathy Questionnaire

**RAAM-PEF**		Veterans Affairs members	68.7±9.1 (control)				Eplerenone 50mg daily from 2 – 24 weeks							
**Edelmann (2013)** **ALDO-DHF**	Germany, Austria	NYHA class II – III Age >50	67±8	≥50	213	209	Spironolactone 25mg daily	Placebo	12 months	SBP, DBP	E/A, E/e’, EDT, LVEDD, LVEF, LVMi, LAVi	NT-proBNP	6MWD, peak VO2, NYHA class	36-Item Short Form Health Survey, Minnesota Living with Heart Failure Questionnaire, Hospital Anxiety and Depression Scale
**Kurrelmeyer (2014)**	United States	NYHA class II – III Women only Chronic ACEI or ARB use	70	≥50	24	24	Spironolactone 25mg daily	Placebo	3, 6 months	NR	E/A, lateral and septal E/e’, EDT, LVMi, LAVi	BNP, PINP, PIIIPNP, CTX	6MWD, NYHA class	Kansas City Cardiomyopathy Questionnaire

**Shah (2015)**<br><br>**TOPCAT substudy**	United States, Russia, Georgia	Age ≥50	67.9±9.5 (MRA)<br><br>70.7±8.4 (control)	≥45	121	118	Spironolactone 15 – 45mg daily	Placebo	12, 18 months	NR	E/A, E/’e (septal), LVMi	NR	NR	NR
**Kosmala (2016)**<br><br>**STRUCTURE**	Australia	NYHA class II - III	67±9	>50	75	75	Spironolactone 25mg daily	Placebo	6 months	NR	E/A, E/e’, EDT, GLS, LVMi, LAVi,	BNP, galectin-3	Peak VO2	NR
**Kosmala (2017)**	Australia	NYHA class II – III	64±8	>50	51	54	Spironolactone 25mg daily	Placebo	6 months	SBP, DBP	E/e’, LAVi, GLS	BNP, galectin-3	Peak VO2, NHYA class	NR
**Upadhya (2017)**	United States	NYHA class II – III<br><br>Elderly	71±1	≥50	42	38	Spironolactone 25mg daily	Placebo	4, 9 months	NR	E/A, lateral and septal E/e’, EDT<br><br>CMR parameters	BNP	6MWD, peak VO2, NYHA class	Minnesota Living with Heart Failure Questionnaire

**McDiarmid (2020)**	United Kingdom	NYHA class II – III	75.1±7.3	>50	27	24	Spironolactone 25mg daily	Untreated	6 months	SBP, DBP	Lateral, septal and mean E' CMR parameters	NT-proBNP, PINP, PIIINP, MMP-3	NR	NR
**Shantsila (2020) IMPRESS-AF**	United Kingdom	Age ≥50 Permanent AF	72.3±7.4	≥55	125	125	Spironolactone 25mg daily	Placebo	2 years	SBP, DBP	E/e'	BNP	6MWD, peak VO2, NYHA class	Minnesota Living with Heart Failure Questionnaire, EuroQol-5 Dimensions

Footnotes: NYHA = New York Heart Association; MRA = mineralocorticoid receptor antagonist; NR = not reported; SBP = systolic blood pressure; DBP = diastolic blood pressure
