# Supplementary material for: Mineralocorticoid Receptor Antagonists in Heart Failure with Preserved Ejection Fraction: A Systematic Review and Meta-Analysis
Source: J Clin Med. 2025 May 21;14(10):3598. doi: 10.3390/jcm14103598 (PMC12112577; doi:10.3390/jcm14103598)
Supplement: Supplementary file 1 [file jcm-14-03598-s001.zip › Table S2 - ROB.pdf]

Table S2: Risk of bias assessment Joanna Briggs Institute of Critical Appraisal Tools Checklist for RCTs

		Mottram 2004		Roongsritong 2005		Mak 2009		Deswal 2011		Edelmann 2013		Kurrelmeyer 2014		Shah 2015		Kosmala 2016		Kosmala 2017		Upadhya 2017		McDiarmid 2020		Shantsila 2020	
		MZ	FF	MZ	FF	MZ	FF	MZ	FF	MZ	FF	MZ	FF	MZ	FF	MZ	FF	MZ	FF	MZ	FF	MZ	FF	MZ	FF
1.	Was true randomization used for assignment of participants to treatment groups?	U	U	U	U	U	U	U	U	U	U	U	U	Y	Y	Y	Y	U	U	U	U	Y	Y	Y	Y
2.	Was allocation to groups concealed?	U	U	U	U	N	N	U	U	U	U	Y	Y	U	U	Y	Y	U	U	Y	Y	U	U	Y	Y
3.	Were treatment groups similar at the baseline?	Y	Y	U	U	Y	Y	U	Y	Y	Y	U	U	Y	Y	Y	Y	Y	Y	Y	Y	Y	Y	Y	Y
4.	Were participants blind to treatment assignment?	Y	Y	Y	Y	N	N	Y	Y	Y	Y	Y	Y	Y	Y	Y	Y	U	U	Y	Y	N	N	Y	Y
5.	Were those delivering treatment blind to	Y	Y	U	U	N	N	N	N	Y	Y	Y	Y	Y	Y	Y	Y	U	U	Y	Y	N	N	Y	Y

	treatment assignment?																								
6.	Were outcomes assessors blind to treatment assignment?	U	U	U	U	Y	Y	Y	Y	U	U	Y	Y	Y	Y	Y	Y	U	U	Y	Y	Y	Y	Y	Y
7.	Were treatment groups treated identically other than the intervention of interest?	Y	Y	U	U	Y	Y	Y	Y	Y	Y	Y	Y	Y	Y	Y	Y	U	U	Y	Y	Y	Y	Y	Y
8.	Was follow up complete and if not, were differences between groups in terms of their follow up adequately described and analyzed?	Y	Y	N	N	N	N	N	N	Y	Y	U	U	Y	Y	Y	Y	Y	Y	Y	Y	Y	U	U	U

9. Were participants analyzed in the groups to which they were randomized?	U	U	Y	Y	Y	Y	Y	Y	Y	Y	Y	Y	Y	Y	Y	Y	Y	Y	Y	Y	Y	Y	Y	Y
10. Were outcomes measured in the same way for treatment groups?	Y	Y	Y	Y	Y	Y	Y	Y	Y	Y	Y	Y	Y	Y	Y	Y	Y	Y	Y	Y	Y	Y	Y	Y
11. Were outcomes measured in a reliable way?	U	U	Y	Y	Y	Y	Y	Y	Y	Y	Y	Y	Y	Y	Y	Y	Y	Y	Y	Y	Y	Y	Y	Y
12. Was appropriate statistical analysis used?	Y	Y	Y	Y	Y	Y	Y	Y	Y	Y	Y	Y	Y	Y	Y	Y	Y	Y	Y	Y	Y	Y	Y	Y
13. Was the trial design appropriate for the topic, and any deviations from the standard RCT design accounted	Y	Y	Y	Y	Y	Y	Y	Y	Y	Y	Y	Y	Y	Y	Y	Y	Y	Y	Y	Y	Y	Y	Y	Y

	for in the conduct and analysis?												
14.	Overall risk of bias	Unclear	Unclear	Unclear	Low	Low	Low	Low	Low	Unclear	Low	Low	Low

Footnotes: Y = yes; N = no; U = unclear.
